# Piezoelectric and Triboelectric Nanogenerators for Enhanced Wound Healing

**DOI:** 10.3390/biomimetics8070517

**Published:** 2023-11-01

**Authors:** Hye-Jeong Jang, Daniel Manaye Tiruneh, Hanjun Ryu, Jeong-Kee Yoon

**Affiliations:** 1 Department of Systems Biotechnology, Chung-Ang University, Anseong-si 17546, Gyeonggi-do, Republic of Korea; misodam0527@gmail.com; 2 Department of Intelligence Energy and Industry, Chung-Ang University, Seoul 06974, Republic of Korea; danishamanua09@gmail.com; 3 Department of Advanced Materials Engineering, Chung-Ang University, Anseong-si 17546, Gyeonggi-do, Republic of Korea

**Keywords:** nanogenerator, piezoelectric, triboelectric, wound healing

## Abstract

Wound healing is a highly orchestrated biological process characterized by sequential phases involving inflammation, proliferation, and tissue remodeling, and the role of endogenous electrical signals in regulating these phases has been highlighted. Recently, external electrostimulation has been shown to enhance these processes by promoting cell migration, extracellular matrix formation, and growth factor release while suppressing pro-inflammatory signals and reducing the risk of infection. Among the innovative approaches, piezoelectric and triboelectric nanogenerators have emerged as the next generation of flexible and wireless electronics designed for energy harvesting and efficiently converting mechanical energy into electrical power. In this review, we discuss recent advances in the emerging field of nanogenerators for harnessing electrical stimulation to accelerate wound healing. We elucidate the fundamental mechanisms of wound healing and relevant bioelectric physiology, as well as the principles underlying each nanogenerator technology, and review their preclinical applications. In addition, we address the prominent challenges and outline the future prospects for this emerging era of electrical wound-healing devices.

## 1. Introduction

Wound healing is a crucial component of human health since it is necessary to treat injuries to the skin or organs. Wounds can take anywhere from days to months to heal, depending on their anatomic location, depth, size and the health condition of the patient. However, inappropriate care of the wound site can lead to serious issues. Impaired wound healing can have catastrophic repercussions in some conditions, such as in venous ulcers, diabetic foot ulcers, and chronic non-healing wounds [1,2,3]. As well as slowing down the healing process, defective wound-healing process can lead to infection, inflammation, and even necrosis, resulting in long-lasting physical and psychological impacts on the patient [4,5,6,7]. In 2019, approximately 10.5 million Medicare beneficiaries in the United States were affected by chronic wounds [8].

With the advancement of biomedical technology, there has been intense research in recent years into new tools and strategies to aid wound healing and to improve the promotion of wound healing in patients. The conventional treatments of wound healing have been limited to wound dressings, surgeries and antibiotics that do not play a direct role in regulating innate cell behavior [9,10]. Additionally, the introduction of growth factors has recently become a potential therapeutic strategy for wound healing [11,12]; however, it possesses remaining drawbacks such as the quick deterioration of bioactive compounds [13]. Given that wounds need constant and regular monitoring by healthcare professionals, the availability of medical facilities and treatment equipment has a remarkable impact on the well-being of patients. To tackle these obstacles, a novel system must fulfill the requirements of intrinsic cellular regulation, maintenance of bioactivity, and accessibility. Thus, novel and self-sustaining wearable nanogenerators utilizing electrical stimulation including piezoelectric nanogenerator (PENG) and triboelectric nanogenerator (TENG) have recently been gaining significant recognition [14,15,16,17].

Electrical stimulation proves to be a competent approach for wound healing as it employs low-voltage electric currents at the site of the injury to enhance wound healing by stimulating cell proliferation and migration [18]. These two types of nanogenerators are promising technologies for future development and commercialization due to their wide range of possible uses, and simpler and low-cost manufacturing processes [19,20]. TENG and PENG-based nanogenerators have the capability to transform human biomechanical activities into electrical energy [21]. Compared with other therapeutic devices, they are comparatively lightweight, suitable for wearing, pliable, and biocompatible [22].

Here, we initially review the fundamental and anatomical features of skin and wound sites, the wound-healing process, and the bioelectric factors in normal and wound skin tissues. This comprehensive understanding is then used to highlight the latest developments in wound-healing technology using PENG and TENG by enhancing or promoting cell proliferation and migration, inflammatory response regulation, and growth factor release. Finally, the difficulties and prospective directions of wound healing with regard to nanogenerator devices are addressed, giving an overview of recent advances and underlining future possibilities for the application of nanogenerator-based wound-healing systems as an essential part of individualized healthcare.

## 2. Wound Healing and Skin Regeneration

Despite the tremendous breakthroughs obtained in the understanding of the biology of wound healing over the past two decades, wound healing is still considered to be one of the most intricate processes in human skin physiology. The healing of wounds involves cellular and molecular activities that include a complex cascade of reactions and interactions between different lineages of cells and mediators. Understanding the anatomical and physiological underpinnings of skin tissue is crucial in comprehending the concept of wound healing.

### 2.1. Skin Anatomy and Physiology

The skin is the biggest organ in the body, accounting for approximately 15 to 20% of body weight [23]. The skin has numerous critical functions, the most important of which is to help protect our bodies from foreign components. The thickness of skin varies based on where it is located and what function it serves. To maintain the body’s temperature and homeostasis, the skin includes multiple appendages. The skin can be divided into three layers, the epidermis, dermis, and hypodermis, based on their various cellular structures and functions.

The epidermis is a stratified squamous epithelial layer that functions as a physical barrier at the surface of the skin [24]. In addition to keratinocytes, it also contains melanocytes, which create melanin, Langerhans cells, which take part in the immunological response, and Merkel cells, which are involved in touch sensitivity [25,26,27]. The epidermis is made up of several layers, including the stratum corneum at the top, the stratum granulosum and stratum spinosum in the middle, and the stratum basale at the bottom [28]. Since the epidermis continually renews itself through cell movement and shedding process from stratum basale up to the outermost stratum corneum, these layers indicate the different phases of epidermal development [29].

The dermis is the layer of skin underneath the epidermis and above the hypodermis which supplies nutrition and mechanical assistance to the epidermis [30]. It consists of a complex network of fibroblasts, vascular networks, lymph vessels, nerve endings, mast cells, hair follicles, and glands [31]. Fibroblasts are the major dermal element that produces extracellular matrix components such as collagen and elastin, providing elasticity and durability to the skin [32]. The majority of the skin weight is composed of the dermis, which helps regulate body temperature and provides protection against mechanical damages [33].

The hypodermis, also known as a subcutaneous tissue, is the skin’s innermost layer, comprised of adipose tissues, blood vessels, and sensory neurons [34]. This layer is crucial for protecting organs and bones, providing energy storage, and regulating the overall body and skin temperature [35].

### 2.2. Wounds

**Figure 1:**
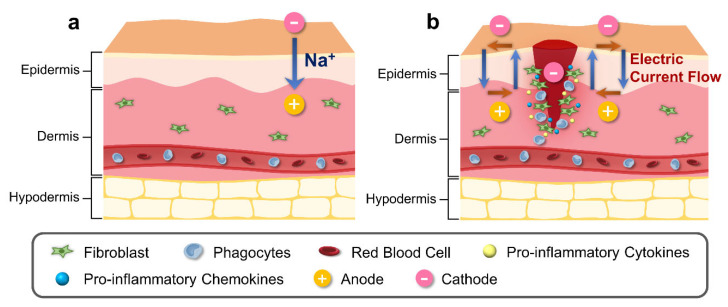
Schematic illustration of physiological and bioelectrical factors of normal and wound skin. (**a**) Electric potential of normal skin is generated by asymmetric ion fluxes across the tissue layers through the Na⁺/K⁺ pump in the epidermis. (**b**) Injury to skin generates cathode in the center of the wound, disrupting the regular current flow. Difference in electric potential between the edges of the wound and the center of the wound creates a current flow that stimulates cell migration to the wound site for increased proliferation and phagocytosis, with release of pro-inflammatory cytokines and chemokines.

Wounds occur when the skin’s inherent integrity is disrupted or damaged, and the scale and degree of the wound may differ significantly. When a wound is not completely healed, the functionality of the skin barrier deteriorates, allowing foreign pathogens to readily penetrate our bodies, which may lead to an infection [36]. Wound healing continues to be a clinical challenge; thus, successful wound treatment is indispensable. The extracellular matrix, various cell types, as well as different growth factors and cytokines are all involved in the wound-healing process (Figure 1b) [37]. Stages of wound healing are commonly classified into four different phases: hemostasis phase, inflammatory phase, proliferation phase, and remodeling phase.

### 2.3. Process of Wound Healing

#### 2.3.1. Hemostasis Phase

When a skin injury occurs, bleeding normally ensues, which initiates hemostasis [38]. To close an opening in blood vessels, platelets start to collect at the wound lesion by binding to the sub-endothelial surface [39]. Following the formation of a mesh-like structure between the fibrin and the platelets, prothrombin is then produced for thickening the blood’s consistency [40]. This procedure creates a fibrin clot, which serves as a scaffold for the inflow of keratinocytes and fibroblasts and traps platelets and cells in the wound region [41].

#### 2.3.2. Inflammatory Phase

During the inflammatory phase, the platelet-derived growth factor and transforming growth factor promote the multiplication of fibroblasts [42]. By generating pro-inflammatory cytokines and chemokines including IL-1, IL-6, IL-10, TNF-α, IL-1β, IFN-γ, CCL2, and CXCL5, these activated fibroblasts participate in a crosstalk that enhances the immune cell activation [43,44]. Monocytes, endothelial cells, and neutrophils attach to the fibrin scaffold generated during platelet activation [38,45]. In order to avoid additional infection and prepare for tissue regeneration at wound sites, neutrophils commence phagocytosis of pathogens and cellular waste [46]. Heat, swelling, redness, pain, and functional loss at the wound areas are the main characteristics of the inflammatory phase [47,48,49].

#### 2.3.3. Proliferation Phase

During the proliferation phase, fibroblasts begin to migrate toward the wound site and proliferate, elevating fibroblast cell density and increasing type III collagen production, while myofibroblasts pull wound edges while bringing them together via contraction [50,51,52]. New blood vessels for enhanced supply of oxygen are formed, along with epithelial cell migration towards the wound region, forming a barrier and repairing the integrity of the defective area [53]. Cells also commence releasing different growth factors, including as transforming growth factor-beta (TGF-β), insulin like growth factor-1 (IGF-1), keratinocyte growth factor-1 (KGF-1), fibroblast growth factor-2 (FGF-2), vascular endothelial growth factor (VEGF), and platelet-derived growth factor (PDGF), that promote the regeneration of new tissue and extracellular matrix as the population of macrophages in the wound site starts to decline [54].

#### 2.3.4. Remodeling Phase

The transformation of granulation tissue into connective tissue is the ultimate stage of wound healing [55]. TGF-β plays an important role in the early stages of the remodeling phase by restricting collagen breakdown as well as stimulating wound contraction by activating metalloproteinases [56]. Furthermore, TGF-β also triggers fibroblasts to transform into myofibroblasts for granulation tissue development [57]. For increased elasticity, type III collagen is substituted with type I collagen; however, impairment in this transition process might result in excessive scar development [58]. Procollagen, the precursor form of collagen, is initially produced; it is then converted into fibers that align parallel to one another and are cross-linked to create more robust strands [59]. Apoptosis and cell maturation of fibroblasts and macrophages alter the formation of connective tissue, while collagen and ECM tissues are continually being loaded into the wound region [60,61].

## 3. Bioelectrical Factors in Skin Tissue

The skin demonstrates complicated electrical characteristics that involve physiological and physical components in terms of its physical and chemical condition [62]. The electrical features of the skin are of significant importance in bioengineering applications given that physical attributes such as impedance and conductance reflect the composition of the skin. Recent developments in the study of the electrical aspects of skin are broadening the spectrum of applications of the technologies for assessing and evaluating skin barrier integrity and the wound-healing process. In this chapter, we focus on the electrical characteristics and functions of normal skin and skin during wound healing.

### 3.1. Bioelectrical Properties in Normal Skin

The electrical characteristics of normal skin are the primary focus of bioimpedance research. Ions are the major charge carrier in biological materials [63]. The electrical conductivity of skin tissue is determined by multiple factors, including electric field frequency, tissue structure, charge carrier mobility, and is regulated by electrons and ions [64,65].

#### 3.1.1. Epidermal Ion Distribution

The skin contains an inherent bioelectric system that generates endogenous electrochemical signals [66]. Electrical potential energy is produced by asymmetrical ionic fluxes across tissue layers of the skin via the sodium–potassium pump in the epidermis (Figure 1a) [67]. Due to the tightly connected keratinocytes comprising the epidermis, there is a higher level of ion transport within cells and less diffusion in the surrounding tissues [68]. Several studies have indicated the presence of divalent ions including calcium and magnesium, which exhibit noticeable peaks at the boundary between the epidermis and the stratum corneum [69,70]. Sodium and chloride are dispersed uniformly throughout the epidermis; however, potassium levels decline significantly from the stratum granulosum to the bottom layers [71]. Calcium has been identified as a significant signaling ion, as its distribution aligns with the distinct requirements of each skin layer at individual differentiation stages. Low calcium concentrations in deeper skin layers stimulate proliferative activity, while high concentrations near top layers differentiate and secrete lamellar bodies, thereby creating a functional skin barrier [72,73].

#### 3.1.2. Transepithelial Potential (TEP)

The gradient of ions across the skin tissue creates a potential difference called the transepithelial potential (TEP), also known as the ‘skin battery’, along the depth of the epidermis [74]. The voltage difference between the stratum corneum and the dermis in humans has been reported to average −23 mV, with variations depending on anatomical location [75]. Early research centered on the connection across different electrical properties of the skin along with the psychological reaction being relevant to sweat gland activity [76,77]. Subsequently, a potential difference produced independently across the epidermis was identified, which stimulated the attention of researchers in the involvement of ion transport in the electrophysiological system of epidermal keratinocytes. TEP values ranging from 10 to 60 mV have been measured on intact epidermis; however, due to the relatively low number of tissues under the dermis, this results in weakly retained ionic gradients and a small potential difference [78,79].

#### 3.1.3. Bioimpedance and Barrier Properties of Skin

The stratum corneum influences the overall skin impedance mainly through its hydrophobic properties, which act as a permeability barrier, as the applied current is conducted through the skin tissue by the water-soluble charged molecular components present [80]. Multi-layered skin models are often described as a mixture of comparable circuits composed of living tissues associated with ionic molecules and their motilities [81]. The impedance properties are essential for effective biomimetic design in terms of transdermal monitoring of current flow.

Like many other biological tissues, the skin displays dielectric characteristics [82]. When dealing with low frequencies, the skin exhibits an increased resistance, while at higher frequencies, it is reduced due to its capacitive properties [83,84]. Additionally, applying high voltage pulses on skin tissue can cause electroporation, which increases cell permeability, as well as electroosmotic flow and thermal perturbation, which causes a temporary or permanent shift in molecular dynamics, boosting skin conductivity [85,86].

### 3.2. Bioelectrical Factors during Wound Healing

#### 3.2.1. Alteration in Epidermal Ion Distribution and Electric Potential

The ionic composition of the skin has been observed to be influenced by the infiltration of exogenous substances [87]. In normal skin, the epidermal electrical potential is generated by asymmetric ion fluxes that continuously pump Na⁺ ions within the tissue layers via the Na⁺/K⁺ pump (Figure 1b). However, during the process of wound healing or skin injury, the current generated from the skin undergoes alteration (Figure 1b). During the process of wound healing or skin injury, the current generated from the skin undergoes alteration (Figure 1b). The electrical potential created by the wounded skin, which is directed towards the area of the wound and has a cathode in the center and an anode at the edge, attracts cells to the injured area [88]. This potential is created by the wounded skin’s current, and the longitudinal electric field created by this current will last until the resistance rises throughout the healing process [89]. The variables that influence TEP and ion distribution following a temporary wound or skin barrier disruption subsequently return to normal levels through a combination of passive diffusion and active transport in the tissue during wound healing [90].

When calcium levels in the stratum granulosum are diminished, barrier restoration mechanisms are activated, which involves the secretion of lamellar bodies into the intercellular gaps of the stratum corneum to replenish them with lipids [72]. Furthermore, calcium also plays a crucial role in the formation of desmosomes and migration of cells in the wound-healing process [91]. In the early phases of wound healing, desmosomal adhesion is regulated in a calcium-dependent manner permitting the cell movement, and over time, providing mechanical support for the regenerating epithelium [92].

#### 3.2.2. Electric Field Profiles in Wound Sites

One of early studies utilized a galvanometer to measure roughly 1 µA emanating from a cut in a finger, which gave rise to current research on an electric field resulting from a wound [93]. In another study of human fingertip amputation currents, the vibrating probe technique was used to measure the leakage current across epithelial tissue in low-resistance regions, with a maximum value of up to 30 A/cm^2^ [94]. In a different study, a vibrating probe was used to measure the lateral electric field of a lancet wound [95]. The reported results showed that the electric field in 18- to 25-year-olds was on average 107 to 148 mV/mm, which was 48% larger than that in 65- to 80-year-olds, demonstrating an age-related influence on the electric field within the region between the epidermis and stratum corneum. [95]. In a mouse wound model, the mean value of the lateral electric field immediately after wound formation was 122 ± 9 mV/mm, and after the wound site was filled with organized and dense epidermal layers, the mean value decreased to 59 ± 5 mV/mm, suggesting that the variance in electrical surface potential is greater in wounds and decreases gradually as the skin returns to its normal state [95].

#### 3.2.3. Effects of Electric Field Alteration on Cell Components

Keratinocytes move towards the cathode at a speed of approximately 1 µm/min when subjected to electric fields of 100 mV/mm [78,96]. In vitro, a direct current of 200 mV/mm, which is comparable to the electrophysiological field strength of skin wounds, attracts keratinocytes to the cathode pole and causes autophagy [97]. Keratinocytes are also propelled toward the cathode by a pulsed electric stimulation of at least 150 mV/mm independently of the frequency of the pulses [98]. Another study employing an electric field of 50 mV/mm, reported the migration of keratinocytes in the direction of the anode [99]. This observation of keratinocyte migration to the anode appears to contradict previous findings and may be due to differences in keratinocyte integrin expression, an important factor in wound healing as integrins are involved in the regulation of keratinocyte functions [100]. The axial positions of fibroblasts are parallel to the electric field [101,102]. When fibroblasts are exposed to an electric field of at least 100 mV/mm, collagen synthesis accelerates by approximately 100% and DNA synthesis enhances by 20%, resulting in efficient wound healing [103]. Furthermore, a physiological electrodynamic field attracts macrophages to the anode pole, increasing their capacity for phagocytosis, whereas their progenitor, the monocyte, is drawn to the cathode pole [104]. Thus, electric fields are thought to play a significant role in the overall wound-healing process through their effects on cell migration and the immune system.

## 4. Nanogenerators for Tissue Regeneration

### 4.1. Types and Principles of Nanogenerators

**Figure 2:**
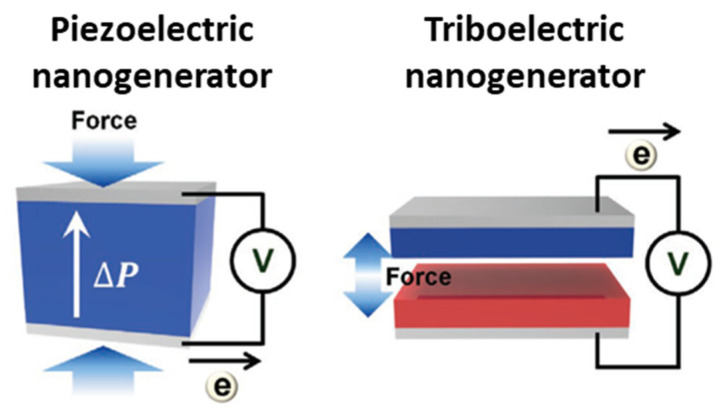
Schematic image of piezoelectric nanogenerator and triboelectric nanogenerator [113]. Reprinted with permission from Ryu et al., Copyright © 2019 John Wiley & Sons, Inc.

PENG [14,15,105] and TENG [16,17,106,107] are promising technologies that convert mechanical energy into electricity (Figure 2). The piezoelectric effect is the result of a transient change in the aligned polarization within a crystalline structure (e.g., ZnO [108], PZT [109], BTO [110], P(VDF-TrFE) [111], and lead-free materials such as LiBnO_3_ [112]) via an external force. Mechanical-strain-induced crystal structure deformation produces internal electric field changes and dipole polarization changes in piezoelectric materials. This phenomenon is a result of the unique asymmetry in the crystal structure of these materials. When mechanical stress is applied, it distorts the crystal lattice, creating an electric potential difference. The piezoelectric potential that is generated by inherent polarization induces the movement of electrons and holes between the top and bottom of the piezoelectric materials, respectively. The piezoelectric potential changes, whether induced by inherent polarization or an external force, are attributed to the flow of electrons between the electrodes. After the external force is removed, the piezoelectric potential returns to its original state, and the electrons flow back. These bidirectional electron flows generate alternating current (AC) electricity, a phenomenon that can be harnessed for various applications. High piezoelectric constant materials such as PZT are known for their efficiency in converting mechanical energy into electricity.

The contact electrostatic effect between two different materials via contact and separation generates triboelectric charges on the materials. This effect relies on the differences in electron affinity between the materials. During contact, one material, known as the positive triboelectric material, acquires positive triboelectric surface charges, while the other, as the negative triboelectric material, accumulates negative triboelectric surface charges [113]. These surface charges initiate the current flow between the material electrodes to compensate for the surface potential difference. Upon contact, surface charges neutralize each other, resulting in a lack of excess electrons and holes on their electrodes. When these materials separate due to an external force, positive and negative triboelectric surface charges induce electrons and holes to compensate for the surface potential, generating a reverse current flow. Consequently, TENGs can efficiently generate alternating current (AC) electricity from the mechanical process of contact and separation. Triboelectric nanogenerators provide a broad range of material options because these devices can be made from almost any material, including paper, metals, and polymers [114]. Positive triboelectric materials are usually selected as metals due to the flow of free electrons and negative triboelectric materials being fluorine polymers, which have high electronegativity to maximize electron attraction from the positive triboelectric materials.

### 4.2. Applications of Nanogenerators for Enhanced Wound Healing

**Figure 3:**
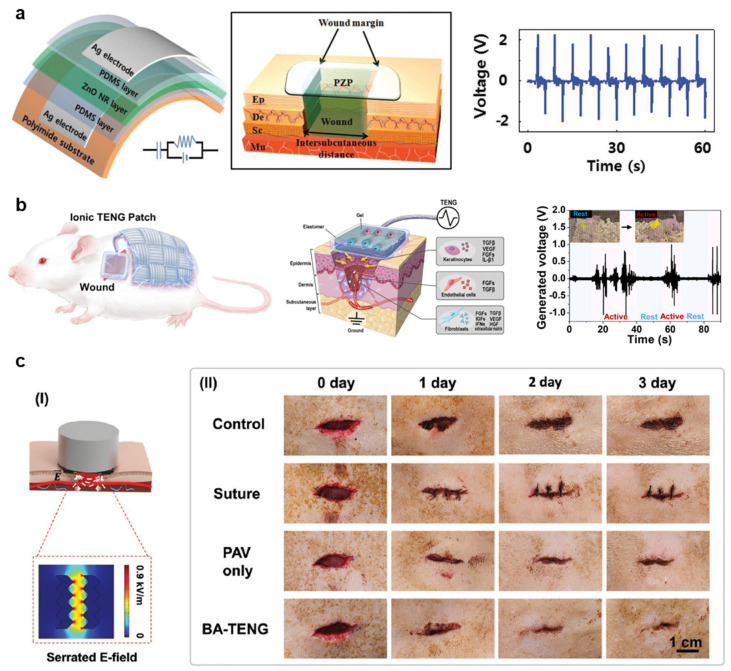
(**a**) Schematic illustrations and output voltage of ZnO-based piezoelectric wound patch [115]. Reprinted with permission from Bhang et al., Copyright © 2016 John Wiley & Sons, Inc. (**b**) Schematic illustrations and output voltage of triboelectric nanogenerator for wound patch [117]. Reprinted with permission from Jeong et al., Copyright © 2021 Elsevier Ltd. (**c**) (I) Schematic representation of ultrasound-driven triboelectric nanogenerator. (II) In vivo experimental results to evaluate the wound-healing effect of BA-TENG [118]. Reprinted with permission from Meng et al., Copyright © 2023 John Wiley & Sons, Inc.

Zinc-oxide-nanorods (ZnO NR)-based piezoelectric dermal patch (PZP) demonstrates electrostimulation for wound healing (Figure 3a) [115]. Bidirectionally grown ZnO NRs using the hydrothermal method have a length and diameter of 2.79 ± 0.14 and 0.58 ± 0.07 μm, respectively. ZnO NRs on polydimethylsiloxane (PDMS) are aligned by rubbing in one direction. PZP generates 1.8 V and 85 nA/cm^2^ via a bending radius of 5 mm. In vitro studies confirm that an exogenous electric field increases dermal fibroblast cell proliferation and enhances myofibroblastic differentiation while regulating inflammation on the wound site. In addition, electrostimulation promotes dermal fibroblast migration and enhances fibroblast growth factor (FGF-2), and the expressions of transforming growth factor-β (TGF-β), receptor (TGFβR), and collagen type III (COL III). A square shape of a full-thickness excisional wound (1.8 × 1.8 cm^2^) on female athymic mice with electrostimulation via the PZP confirms the electrostimulation-induced wound-healing effect. The PZP generates 0.9 V during in vivo studies due to small bending caused by animal behavior within an appropriate range of electrical voltage to promote wound healing, from 150 to 1200 mV [116], and the output performance of the PZP maintains over 15 days without degradation. In vivo studies demonstrate that electrostimulation significantly enhances the wound-healing process, and regenerates the basal layer, and epidermis layer. The Western blot analysis reveals that electrostimulation activates intercellular signaling pathways. For example, phosphorylation of protein kinase B (Akt), phosphoinositide 3-kinase (PI3K), and extracellular signal-regulated kinases (ERK1/2) are polarized along with cell migration.

Poly(vinylidene fluoride) (PVDF), one of the ferroelectric polymers, is widely utilized for flexible PENG applications. Here, the PVDF nanofibers achieved using the electrospinning technique realize a flexible PENG to promote the wound-healing process [119]. The PVDF nanofibers with a polydopamine–polyacrylamide (PDA-PAAm) hydrogel matrix easily adhere to the skin. Hydrogel has a tensile strength of ~15 kPa, elongation of ~500%, Young’s modulus of 46 kPa, adhesion strength of ~5 kPa, and peeling energy of ~45 J/m^2^. The output voltage and current of the PENG are ~0.85 V and 40 nA, respectively, and maintain their performance over 5 month. The in vitro cytocompatibility test confirms that the PVDF flexible PENG exhibits good cytocompatibility. Electrical stimulation increases cell proliferation and migration by 12% compared to controls after 3 days. The cellular scratch healing assay shows a healing area ~1.6 times larger than that of the control group, which indicates that electrostimulation promotes cell migration. In vivo studies demonstrate biomechanical energy-driven electrostimulation on a circular full-thickness skin wound (diameter in 5 mm); the generated voltage is 0.1–0.5 V. The experimental group completely closes the wound in 10 days with a reduced inflammatory response, but the control group has ~23% of an unhealed wound; the control group takes 15 days for complete wound closing. Several biomarkers such as CD31 (endothelial cell marker), VEGF-A (growth factor for angiogenesis) and TGF-β1 (growth factor for tissue regeneration) show a significantly higher presence in the dermis after 6 days of electrostimulation. Weight, hematology analysis, and blood chemistry confirm the device has good biocompatibility. A recent study produced elastic piezoelectric membranes made of PVDF-TrFE(trifluoroethylene)/barium titanate composites [120]. Ultrasound stimulation of piezoelectric membranes stimulated fibroblast proliferation and migration, resulting in enhanced wound healing in a mouse model. In another research, a piezoelectric PVDF/sodium alginate (SA) hydrogel scaffold was created and reinforced with ZnO nanoparticles using a 3D printing technique [121]. This scaffold, with its piezoelectric response, accelerated wound healing through its antimicrobial and anti-inflammatory effects. Another study suggested a combined wearable nanogenerator that utilizes piezoelectric technology with an electro-responsive hydrogel that contains an inhibitor for phosphatase and tensin homologue (PTEN) [122]. By effectively inhibiting PTEN using PENG, they demonstrated enhanced wound healing due to the heightened responsiveness of endothelial cells to electric stimuli.

The example of a PENGs-based system demonstrates tens of nA for enhancing wound healing. Since its current is very small, studying higher current levels may be important. Textile-type wearable ionic TENG (iTENG) offers a fully stretchable system and coverage for infection prevention for small animal experiments to minimize the disruption of animal normal behaviors (Figure 3b) [117]. Organogel-filled silicon rubber microtubes that contain lithium chloride (LiCl) salts for ionic conductivity and stretchability, facilitate reliable structural reconfigurability and durability. The fabrication of ionic fabric maximizes the contact electrification area with the skin, and surface heptadecafluoro-1,1,2,2-tetrahydrodecyl trichlorosilane (HDFS) treatment enhances triboelectric negative properties. The iTENG on a finger generates 0.75–3.3 V_pp_ through a bending angle from 30° to 90°, respectively. Vertical contact and separation with aluminum (Al) plates can generate 25–75 V_pp_, 3.6 μA, and 4.1 μW/cm^2^. Electrostimulation promotes the migration of human dermal fibroblasts and diabetes mellitus by 3.5 and 1.8 times, respectively. ELISA (Enzyme-Linked Immunosorbent Assay) confirms that electrostimulation reactivates fibroblast activity, enhances angiogenesis, and accelerates re-epithelialization. Real-time in vivo analysis confirms that iTENG generates up to 2 V_pp_ when a nude mouse behaves in a cage. Quantification analysis of wound size reveals that electrostimulation accelerated the wound-healing process by ~20%. Histological analysis shows that the electrosimulation group has a thicker epidermis (~100 μm thick) than the control group (~70 μm thick) and 2~3 times higher collagen density. In another study, a wearable TENG wound dressing was developed to promote wound closure through antimicrobial properties, improved fibroblast proliferation and migration, and angiogenesis [123]. A different study developed a bioresorbable TENG-based stimulator using a 3D printing method, and demonstrated improved wound healing in TENG-stimulated rat wound models [124].

In a different study, a TENG-based intrinsically bactericidal device with greater flexibility, ventilation, and moisture retention was developed [125]. Initially, polycaprolactone (PCL) and poly(lactic-co-glycolic acid) (PLGA) membranes were prepared via an electrospinning method; subsequently, a polypyrrole (PPY) layer was applied via chemical vapor deposition onto the electrospun PCL membrane, and finally a stacked structural device was formed from these three membranes [125]. Once assembled, the PPY/PCL membrane demonstrates significant electrical conductivity and elongation at a break of more than 400% [125]. Electrical stimulation and induced positive charges on the PPY surface eradicated more than 96% of bacteria by disrupting the cell membrane [125]. Furthermore, the TENG-based patch accelerated the healing process of wounds in an infected diabetic rat model within 14 days, which highlights the importance of PPY as a TENG electrode material, addressing the potential as an alternative for antibiotics or metallic components [125]. In vitro and in vivo studies both indicated that the applied electrical stimulation on wound sites improved the expression level of genes encoding the VEGF, thus accelerating the process of wound healing [125].

Another example of bioadhesive TENG (BA-TENG, 1.5 cm × 1.5 cm dimensions) demonstrates a flexible and biocompatible TENG that can adhere to wet tissue (Figure 3c) [118]. Acoustic impedance differences between the metal and polymers initiate to vibrate polymer triboelectric layer. PCL-based polyurethane (PCL-r-PU), including autonomous functional urethane (-NH-(C=O)-O-) groups, is electron-rich positive triboelectric; poly(3,4-ethylene dioxythiophene) polystyrene sulfonate (PEDOT:PSS) deposit on PCL-r-PU is a triboelectric electrode. N-hydroxysuccinimideester (PAA-NHS)–poly(vinyl alcohol) copolymer (PAV) is utilized as a bio-adhesive. Molybdenum (Mo) electrodes connect to a wound for electrostimulation; one Mo electrode connects to PEDOT:PSS and another electrode is parallelly freestanding on tissue as a ground. Ultrasound of 20 kHz vibrates the PCL-r-PU membrane and creates friction between PCL-r-PU and PEDOT:PSS/PCL-r-PU. The output voltage and current are 0.82–1.5 V and 6.5–24.2 μA at an ultrasound power density of 0.5–1.0 W/cm^2^, respectively. BA-TENG demonstrates strong adhesion to wet isolated porcine colon with a 5 mm diameter defect in 5 s due to covalent amide bonding and hydrogen bonding between PAV and tissue; interfacial toughness is 150 J/m^2^ and shear strength is 40 kPa. The cytotoxicity test using fibroblast cells confirms that BA-TENG has good biocompatibility. In vivo studies using the Sprague Dawley rat confirm that the BA-TENG effectively blocks bleeding liver injury in 5 s through enhanced hemostasis. Applying ultrasonic on the BA-TENG generates an electric field strength of 0.86 kV/m between Mo electrodes. Serrated-shape electrodes present a tip-enhanced electric field. In vitro tests using fibroblast cells show enhanced cell migration behavior under electrical stimulations. The control and experimental groups cover 47% and 25%, respectively, in 16 h. After 48 h, the electrostimulation group shows ~1.7 times higher cell density than the control group. In vivo studies also exhibit a similar tendency like in vitro test results. The electrostimulation group demonstrates wound closing (1.5 cm × 0.5 cm wound) in 3 days. Thus, electrostimulation using nanogenerators for wound regeneration is a fascinating methodology for next-generation wound care. An additional study fabricated an electronic skin patch based on TENG technology and exhibited improved formation of collagen, re-epithelization, and angiogenesis, resulting in the faster healing of wounds in mouse models [126]. Another study developed a dressing using a piezoelectrically driven triboelectric nanogenerator (PTENG) made from PVDF with a conductive hydrogel [127]. PTENG dressing showed enhanced cell growth and migration and accelerated wound healing in a rat excisional model.

## 5. Conclusions and Future Perspectives

In this review, we present an overview of wound-healing acceleration via electrostimulation using two types of nanogenerators, PENGs and TENGs, which convert mechanical energy to electrical energy from daily activities. They are self-powered devices or battery-less, utilizing the energy of the human body for their operation, or can be externally driven and controlled whenever needed. They are gaining significant attention in biomedical engineering due to their low cost, flexibility, light weight, and controllability, and are finding applications not only in tissue regeneration but also in biosensors and wearable devices. However, nanogenerator-based wound-healing systems face challenges, including long-term stability issues when attached to wound sites due to potential corrosion caused by body fluids, lower regenerative effects compared with growth factor treatments, and a lack of standardized protocols owing to variations in wound defect sizes among patients. The current research focuses on material and structural optimization for the development of a next-generation device for higher power output. Moreover, researchers are actively addressing the limitations by exploring coating materials for improved long-term stability and prevention from unwanted degradation of the nanogenerator induced by mechanical damage or fatigue stress [128,129]. In addition, the miniaturization of the devices with higher flexibility and transparency has been studied to minimize user discomfort [130,131], as well as the addition of a nanogenerator-based biosensor or display [132,133] for a better user interface. Overall, ongoing research into the development and application of nanogenerators in regenerative medicine holds great promise for advancing this interdisciplinary field and paving the way for precise and effective therapeutic interventions.

## Data Availability

Not applicable.
